# Novel *PIGT* Variant in Two Brothers: Expansion of the Multiple Congenital Anomalies-Hypotonia Seizures Syndrome 3 Phenotype

**DOI:** 10.3390/genes7120108

**Published:** 2016-11-29

**Authors:** Nadia Skauli, Sean Wallace, Samuel C. C. Chiang, Tuva Barøy, Asbjørn Holmgren, Asbjørg Stray-Pedersen, Yenan T. Bryceson, Petter Strømme, Eirik Frengen, Doriana Misceo

**Affiliations:** 1 Department of Medical Genetics, Oslo University Hospital, 0450 Oslo, Norway; nadia.skauli@medisin.uio.no (N.S.); tuva.baroy@medisin.uio.no (T.B.); asbjorn.holmgren@medisin.uio.no.uio.no (A.H.); astraype@ous-hf.no (A.S.-P.); eirik.frengen@medisin.uio.no (E.F.); 2 Faculty of Medicine, University of Oslo, 0450 Oslo, Norway; petter.stromme@medisin.uio.no; 3 Department of Clinical Neurosciences for Children, Division of Pediatric and Adolescent Medicine, Oslo University Hospital, 0450 Oslo, Norway; s.c.wallace@medisin.uio.no; 4 Center for Hematology and Regenerative Medicine (HERM), Department of Medicine, Karolinska University Hospital Huddinge, 14157 Stockholm, Sweden; samuel.chiang-cern-cher@ki.se (S.C.C.C.); yenan.bryceson@ki.se (Y.T.B.)

**Keywords:** cerebellar atrophy, craniofacial dysmorphism, developmental delay, epilepsy, GPI-anchors, MCAHS3, *PIGT*

## Abstract

Biallelic *PIGT* variants were previously reported in seven patients from three families with Multiple Congenital Anomalies-Hypotonia Seizures Syndrome 3 (MCAHS3), characterized by epileptic encephalopathy, hypotonia, global developmental delay/intellectual disability, cerebral and cerebellar atrophy, craniofacial dysmorphisms, and skeletal, ophthalmological, cardiac, and genitourinary abnormalities. We report a novel homozygous *PIGT* missense variant c.1079G>T (p.Gly360Val) in two brothers with several of the typical features of MCAHS3, but in addition, pyramidal tract neurological signs. Notably, they are the first patients with MCAHS3 without skeletal, cardiac, or genitourinary anomalies. *PIGT* encodes a crucial subunit of the glycosylphosphatidylinositol (GPI) transamidase complex, which catalyzes the attachment of proteins to GPI-anchors, attaching the proteins to the cell membrane. In vitro studies in cells from the two brothers showed reduced levels of GPI-anchors and GPI-anchored proteins on the cell surface, supporting the pathogenicity of the novel *PIGT* variant.

## 1. Introduction

More than 150 extracellular proteins in humans are attached to the plasma membrane through a glycosylphosphatidylinositol (GPI) anchor. Roles of GPI anchored proteins (GPI-AP) in the cells are various: signaling, cell adhesion, receptors, protease inhibitors, transcytotic transporters, complement regulatory proteins, and enzymatic functions. The transfer of the GPI-anchor to proteins carrying a *C*-terminal GPI-attachment signal is a post-translational modification catalyzed by the GPI transamidase (GPI-TA) complex [1]. The GPI-TA complex contains several crucial subunits, including the protein encoded by *PIGT*. PIG-T is necessary for the attachment of a GPI-anchor to proteins by the generation of carbonyl intermediates. Furthermore, the GPI-TA complex is dependent on PIG-T for stability by its role in linking PIG-S to GAA1 and GPI8 (GPI-TA complex subunits) [2,3].

Mutations in 12 genes encoding proteins involved in GPI-anchor biosynthesis and GPI-anchor protein modification, including PIG-T, are known to result in GPI-deficiency disorders, typically presenting with drug resistant epilepsy and hypotonia [4,5,6,7,8,9,10,11,12,13,14,15]. Biallelic variants in *PIGT* were previously described in seven patients from three families with Multiple Congenital Anomalies-Hypotonia Seizures Syndrome 3 (MCAHS3, Online Mendelian Inheritance in Man (OMIM) 615398) [11,16,17], characterized by infantile onset of epilepsy, hypotonia, global developmental delay/intellectual disability (ID), craniofacial dysmorphic features, ophthalmological defects, cerebral and cerebellar atrophy, and congenital anomalies involving skeletal, cardiac, and genitourinary systems. We report a novel homozygous *PIGT* missense variant c.1079G>T (p.Gly360Val) in two brothers presenting with infantile onset epilepsy, hypotonia, severe ID, dysmorphic features, ophthalmological defects, and brain dysfunction typical of MCAHS3. In notable contrast to previously reported patients with MCAHS3, they did not manifest skeletal, cardiac, or genitourinary anomalies. They also exhibited pyramidal tract involvement from the age of five years. Flow cytometry studies on cells obtained from the patients were performed to show pathogenicity of the novel *PIGT* variant.

## 2. Experimental Section

### 2.1. Clinical Description

**Figure 1:**
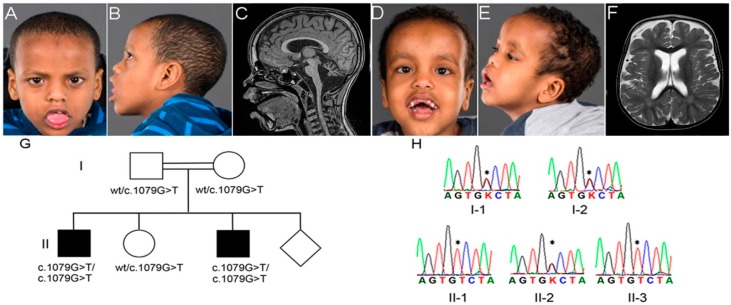
(**A**,**B**) Photographs of patient 1 taken at seven years; and (**C**) cerebral magnetic resonance imaging (MRI) with T1 sagittal midline-view of patient 1 at nine years; (**D**,**E**) Photographs of patient 2 taken at five years; and (**F**) MRI with T2 axial view at the level of centrum semiovale of patient 2 at 14 months; (**G**) Family pedigree with segregation of the *PIGT* c.1079G>T; and (**H**) chromatograms showing the Sanger sequencing of the *PIGT* c.1079G>T in the family members. Both patients had a high anterior hairline, a high and broad forehead with frontal bossing, bitemporal narrowing, a short nose with anteverted nares and a depressed nasal bridge, a short philtrum, a tented lip, and a wide, open mouth with sagging of the lower jaw, consistent with hypotonia. Note hyperplastic gingiva, widely spaced and irregularly shaped and sized teeth. Some teeth were missing due to falls (**D**); The ears were low-set in patient 2 (**D**). Both patients had bilateral esotropia, most notable in patient 2 (**D**); On MRI, cerebellar atrophy, particularly of the vermis, was evident in both patients. The atrophic process also caused a prominent concavity of the tegmental part of the brain stem in patient 1 (**C**); There was widening of the subarachnoidal spaces, especially frontally, as shown in patient 2 (**F**); The *PIGT* c.1079G>T segregated with the clinical syndrome, as the two affected brothers were homozygous (II-1 and II-3); and the healthy parents and sister were heterozygous (I-1, I-2, and II-2); wt, wild type (**G**). Chromatograms of the Sanger sequencing, showing the homozygous *PIGT* c.1079G>T variant in the two affected brothers and the heterozygous variant in the healthy parents and the sister. “*” = c.1079G>T; K = G or T (**H**).

Patient 1 (Figure 1A,B) was a nine-year-old boy, born to healthy first-degree cousins of Somalian origin. He was born at term, following an uncomplicated pregnancy, with birth weight at the 50th centile and head circumference (HC) at the 10th centile; length was unknown. He experienced five generalized seizures associated with febrile illness between 6 and 18 months. At the age of six months, hypotonia and developmental delay were evident. Psychomotor regression became obvious at the time of seizure onset at six months: he lost babbling skills, the ability to lift his head when lying on his stomach, and the ability to roll over to his back. After the period of regression, the psychomotor development was slow. He had onset of myoclonic seizures around 12 months of age, rapidly worsening with almost continuous myoclonic seizures (parents reported up to 25 myoclonic seizures daily). There were generalized, myoclonic, tonic, and complex partial seizures. Despite combinations of up to 11 antiepileptic drugs, and a period on the ketogenic diet, the seizures were poorly controlled. The initial electroencephalogram (EEG) performed at six months was normal. When repeated at 12 months, the EEG showed multiple bilateral outbursts of spike-wave epileptiform activity, compatible with idiopathic generalized epilepsy. Later recordings showed generalized epileptic activity that correlated with clinical seizures. Testing with the Bayley scale of Infant and Toddler Development III Edition [18] at 3 years and 7 months showed a cognitive level of 9 months, and language skills of 10–12 months, suggesting severe ID (IQ 20–34). However, he smiled, gave fleeting eye contact, and appeared socially connected. Informal testing by a clinical psychologist at seven years also concluded that he had severe ID. At this age he could use about 15 signs for communication.

A brain magnetic resonance imaging (MRI) examination at nine months showed widening of the subarachnoid spaces that could suggest atrophy of the cerebral cortex (especially frontally) and of the cerebellar vermis and cerebellar hemispheres. Another brain MRI examination at age two years eight months showed unchanged widening of the subarachnoid spaces, but progression of cerebellar atrophy. A third brain MRI at age nine years in addition to previous findings showed prominent concavity of the tegmental part of the brain stem (Figure 1C).

The progression of the cerebellar atrophy correlated with marked truncal and limb ataxia, which became particularly obvious from three years of age. At three and a half years he could sit, crawl, and pull himself up to stand with support, but still had poor head control. He used a helmet with teeth protection due to seizure-associated falls and poor motor coordination. His fine motor skills were inhibited by ataxia and made independent feeding difficult, and eventually a gastrostomy tube was inserted.

From the age of five, pyramidal tract involvement was evident with spasticity of the lower limbs, increased patellar and Achilles reflexes, bilateral ankle clonus, and Babinski sign. Growth was within the normal range until seven years of age, after which weight and height followed curves just below the 2.5th centile, whereas HC was at 10th centile.

Craniofacial dysmorphic features (displayed in Figure 1A,B when the patient was seven years old) were already evident at three years of age: high anterior hairline, high and broad forehead with frontal bossing, bitemporal narrowing, short nose with anteverted nares and depressed nasal bridge, short philtrum, tented lip, and wide, open mouth with sagging of the lower jaw, consistent with hypotonia. Ultrasound examinations of the heart and kidneys were normal. There were no clinical signs of osteopenia, and an X-ray evaluation of the bone age at age nine years six months was normal. Craniosynostosis and pectus excavatum were not present. The parents had no concerns about the child’s hearing; formal testing at the local “health station” some years before was normal. Visual acuity has been difficult to assess due to the patient’s developmental level and reduced cooperation. Following serial testing with Teller acuity cards, however, he is considered to have significantly impaired vision. Cortical visual impairment was regarded to play a part in his visual deficiency. He had vertical gaze nystagmus, astigmatism (refractive error 5.0 diopters right and 2.5 diopters left eye), and esotropia due to inadequate eye abduction (Figure 1A). Surgical correction was not considered, due to the cortical visual impairment. He had normal pupillary reflexes and red reflex. A tendency for inadequate upward gaze was noted. Flash-visual evoked potentials (VEP) and the orbits as seen on cerebral MRI were normal. Electroretinography (ERG) was performed at the age of eight years under anesthesia and revealed a normal scotopic response, while the photopic response was slightly abnormal, but this was not followed up further.

At the age of nine years his psychomotor development appeared severely delayed. He could crawl, but was otherwise sitting in a wheelchair. G-banded karyotype at 550 band resolution and array comparative genome hybridization (aCGH) 105K (Agilent Technologies, Santa Clara, CA, USA) gave normal results.

Patient 2 (Figure 1D,E), the younger brother of patient 1, was a seven-year-old boy, born at term following an uncomplicated pregnancy with birth weight and length at the 50th centile and HC 0.5 cm > 97.5th centile. He had unilateral congenital talipes equinovarus. Hypotonia and developmental delay were noted from three months. The first seizure was triggered by fever at five months. Subsequently, several seizures were also related to fever, however there was a gradual transition to non-febrile generalized tonic-clonic and myoclonic seizures at 11 months, and later also tonic and complex partial seizures. The first EEG at five months was normal. Repeated standard EEGs during the following 10 months demonstrated increased slow-wave activity, but little or no epileptic activity. Like his brother he developed therapy resistant epilepsy.

He showed slow psychomotor development, but did not lose any skills. At 14 months he had no words, but was babbling, gave eye contact, smiled, and appeared socially connected. At two and a half years he could sit, stand with support, and walk some steps with support. He had poor head control, and a relatively large head, which he often kept tilted backwards. At age five, his cognitive level corresponded to 18 months on the Bayley scale of Infant and Toddler Development III Edition [18]. Receptive and expressive language skills tested at the same age with Reynell Developmental Language Scale [19] also corresponded to 18 months. The test results indicated moderate to severe ID.

A brain MRI examination at 14 months showed fluid-filled widening of the subarachnoidal spaces, particularly frontally, suggesting cortical atrophy (Figure 1F), and cerebellar atrophy, primarily affecting the vermis.

Truncal and limb ataxia became evident between three and four years. From age five, he displayed pyramidal tract involvement with spasticity of the lower limbs, increased patellar and Achilles reflexes, bilateral ankle clonus, and Babinski sign. Growth followed curves within the normal ranges: length at the 2.5–10th centiles, weight at the 10–25th centiles, and head circumference at the 75–90th centiles. Craniofacial dysmorphic features at age five years, similar to those of his brother, are shown in Figure 1D,E. There were no clinical signs of osteopenia, and an X-ray evaluation of bone age at age six years and nine months was normal. Hearing was tested at the local health station at 18 months of age and was concluded as normal. Ultrasound examinations of the heart and kidneys were normal.

Like his elder brother he had impaired vision, and limited upward gaze, causing him to tilt his head backwards. He had astigmatism (refractive error 5.25 diopters right and 3.25 diopters left eye), vertical nystagmus, and mildly reduced eye abduction causing esotropia (Figure 1D). Esotropia was not attempted to be addressed surgically due to the cortical visual impairment. He had normal pupillary reflexes and normal red reflex, and funduscopic examination was normal. The cause of his vision deficiency was considered to be cortical visual impairment.

At the age of seven his psychomotor development appeared severely delayed. He could not walk, and was mostly sitting in a wheelchair. G-banded karyotype at 550 band resolution was normal, aCGH 44K (Agilent Technologies) identified a duplication in 15q11.2 (chr15:22784523–23085096 bp, hg19), which was found inherited from his healthy mother by 105K Agilent aCGH.

In both patients, biochemical work-up in blood was normal since birth, including total alkaline phosphatase, calcium, phosphate, and thyroid function tests measured in serum. The mean corpuscular volume (MCV) of red blood cells was in the normal high range with values 96 and 93 fL in patient 1 and 2, respectively (reference 76–95 fL). Amino acids, lactate, and glucose levels measured in cerebrospinal fluid (CSF) and plasma were unremarkable, as well as the levels of organic acids and mucopolysaccharides in urine.

### 2.2. Materials and Methods

The study was conducted in accordance with the Declaration of Helsinki, the research project was approved by the Regional Ethical Committee (REK 2010/1152-1). The parents signed a written informed consent for the genetic analyses, and publication of the results and photos.

#### 2.2.1. Whole Exome Sequencing

Whole Exome Sequencing (WES) was performed on genomic DNA from the patients and their healthy mother with the Illumina TruSeq Exome Enrichment kit (Illumina Inc., San Diego, CA, USA). The exome libraries were sequenced on Illumina HiSeq2000 (Illumina Inc.), with 100 base pairs (bp) paired-end reads. Genome Analysis Tool Kit (GATK) [20] was used to analyze the WES data and functional annotation was performed with snpEff [21] and Variant Effect Predictor (VEP), using Ensembl release 71 [22]. WES data was filtered and analyzed using FILTUS v.0.99-934 [23]. Variants were removed if they had a high probability of being technical artifacts, as computed by the GATKs “variant quality score recalibration” procedure, occurred with a frequency above 1% in 1000 Genomes Project database 5, NHLBI Exome Sequencing Project Exome Variant Server (www.evs.gs.washington.edu) or in an in-house WES database of 443 individuals, or were predicted to have low impact according to snpEff [21], Polyphen-2 [24] or SIFT [25]. Allele frequency was also assessed in the Exome Aggregate database [26], which contains data from more than 60,000 human exomes. *PIGT* exon 9 (chr20:44049937–44050390 bp, hg19) was polymerase chain reaction (PCR) amplified from genomic DNA and sequenced using forward primer 5′-GGGACTCTGAACATGGCCTGGG-3′ and reverse primer 5′-CACAGTGAGGCTCCCGTTCCA-3′. Amplified PCR products were sequenced on ABI 3730 genetic analyzer (Applied Biosystems, Foster City, CA, USA) using standard protocols and analyzed with Sequencing Analysis software (Applied Biosystems) and compared to the *PIGT* reference sequence (NM_015937.5).

#### 2.2.2. Flow Cytometry

Flow cytometry was performed on blood cells from patient 1 and two controls, and skin fibroblasts obtained from both patients and two controls. Blood cells from patient 1 were stained with the following lineage markers and antibodies: CD19 (clone HIB19), and CD45 (Clone HI30) (both purchased from BD Bioscience, San Jose, CA, USA) as well as antibodies against the following GPI-APs: CD14 (M5E2), CD16 (3G8), CD24 (ML5), CD48 (MEM-102), and fluorochrome conjugated aerolysin (FLAER) (Cedarlane, Burlington, NC, USA), which specifically binds GPI-anchors. Isotype control antibodies were used as negative control. Red blood cells were lysed with FACS Lysing Solution (BD Bioscience) and cells acquired on a Fortessa cell analyzer (BD Bioscience). Skin fibroblasts obtained from both patients and healthy controls were cultured in 1× Dulbecco’s Modified Eagle Medium (DMEM) containing 25 mM l-glutamine HEPES (Gibco, Life Technologies, Carlsbad, CA, USA) supplemented with 2% penicillin-streptomycin 10% fetal bovine serum (FBS) and detached by cell scraping before staining with antibodies against GPI-APs CD49 (H19), CD90 (5E10), and FLAER, or corresponding isotype controls. The cells were fixed (Cytofix, BD Bioscience) and acquired on a LSR Fortessa cell analyzer (BD Bioscience) and the median fluorescence intensities (MFI) of the GP-APs in the cell populations were determined and analyzed with FlowJo LLC v9.8 (FlowJo LLC, Ashland, OR, USA).

## 3. Results

### 3.1. Genetic Analysis

WES was performed on DNA from patient 1 and 2, and their mother (II-1, II-3, and I-2, Figure 1G), and the WES data were analyzed according to recessive mode of inheritance based on the family history. Variants after filtering are listed in Supplementary Table S1. Most relevant was a novel homozygous c.1079G>T (p.Gly360Val) variant in *PIGT* (NM_015937.5). This variant was confirmed by Sanger sequencing to segregate with the disease in the family, as both patients (II-1 and II-3) were homozygous, and the healthy parents and sister (I-1, I-2, and II-2) were heterozygous (Figure 1G–H). The *PIGT* variant was predicted to be probably damaging by PolyPhen-2 [24] (score 1.0) and damaging by SIFT [24] (score 0), and it was not seen in the 1000 Genomes database [27], the ExAC database [26] or in our in-house database of 443 exomes of mixed ethnicity. The *PIGT* c.1079G>T alters an evolutionary highly conserved amino acid p.Gly360. It is located in the GPI-TA domain (spanning amino acids 22 to 578) [28], which transfers mature GPI-anchors to target proteins. Biallelic mutations in *PIGT* are known to cause MCAHS3 [11,16,17], which was compatible with the clinical phenotype in the two brothers.

### 3.2. Flow Cytometry

**Figure 2:**
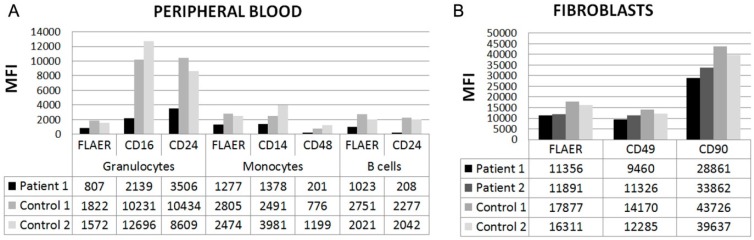
(**A**) Abundance of glycosylphosphatidylinositol (GPI)-anchored proteins measured by flow cytometry in two controls, in patient 1 in peripheral blood, and in patient 1 and 2 in skin fibroblasts (**B**). Cells were stained with fluorochrome conjugated aerolysin (FLAER) to detect total GPI-anchors and with fluorochrome conjugated antibodies to detect GPI-anchored proteins (CD14, CD16, CD24, CD48, CD49, and CD90). The *y*-axis displays Median Fluorescent Intensities (MFI) of proteins on indicated cell populations.

In order to investigate whether the novel *PIGT* variant resulted in a functional defect, the abundance of GPI-anchored proteins and GPI-anchors were studied in patient cells by flow cytometry. Fluorochrome conjugated antibodies were used to measure the levels of the GPI-anchored proteins CD14, CD16, CD24, CD48, CD49, and CD90 in subsets of patient leucocytes or skin fibroblasts compared to the levels in healthy controls. In granulocytes from peripheral blood (patient 1), the median levels of CD16 and CD24 were 19% and 37% compared to controls, the levels of CD14 and CD48 in monocytes were 43% and 20% compared to controls, and the level of CD24 in B-cells was 10% compared to controls (Figure 2A). The levels of CD49 and CD90 in skin fibroblasts from the two patients were only mildly reduced, to about 79% and 75% compared to controls (Figure 2B). In addition, in leukocyte subsets from patient 1, the total levels of GPI-anchors, stained with the GPI-specific marker FLAER, were reduced to less than 50% compared to controls, and the average level of FLAER in fibroblasts from both patients was 68% compared to controls (Figure 2A,B).

## 4. Discussion

Including the present report, nine patients from four families were hitherto reported with MCAHS3, caused by *PIGT* mutations (Table 1) [11,16,17]. Hypotonia, global developmental delay/ID, epileptic seizures, cortical visual impairment, and craniofacial dysmorphisms were present in all patients. Most of the patients had onset of febrile-induced seizures between four and six months of age, which is earlier than the typical age-range. The febrile-induced seizures were subsequently followed by unprovoked and poorly controlled seizures. In general, EEG recordings were normal early in life, and showed epileptic discharges later on. Cerebral cortical and cerebellar atrophy detected by MRI, present in seven patients, were also key features. Similar to one of the previous patients [17], the atrophy displayed in patient 1 in the present report also affected the brain stem. In another patient, the atrophy also affected the basal ganglia [11]. Repeated MRI examinations revealed that the atrophic process, especially of the cerebellum, was most pronounced at a young age [11,16].

A recognizable facial dysmorphic pattern in MCAHS3 includes a high forehead, frontal bossing, bitemporal narrowing, a short nose with anteverted nares and depressed nasal bridge, and a wide, open mouth, consistent with hypotonia (Table 1).

Five of the patients with MCAHS3 had low total levels of the four GPI-linked alkaline phosphatase isoenzymes measured in serum [11,17]. Mutations in *ALPL*, encoding the tissue nonspecific alkaline phosphatase liver/bone/kidney (ALPL) isoenzyme, cause Hypophosphatasia (OMIM 241510). Hypophosphatasia presents with skeletal defects, including bone hypomineralization, likely resulting from extracellular accumulation of the substrate inorganic pyrophosphate (PPi), which is a potent inhibitor of bone mineralization [29,30]. Patients with hypophosphatasia can also manifest epileptic seizures [30,31]. Thus, reduced GPI-anchoring of ALPL was proposed to cause the skeletal defects and epilepsy in the patients with MCAHS3 [11]. However, a normal level of serum alkaline phosphatase was measured in the two brothers in the current report, and in the siblings reported by Lam [16], who presented with both epilepsy and skeletal defects. These findings indicate that a low serum level of the alkaline phosphatase isoenzymes, including ALPL, cannot entirely explain the presence of these clinical features in the patients with MCAHS3.

Patient 1 and 2 exhibited severe affliction of the central nervous system typical of MCAHS3, but also pyramidal tract signs, which were not reported in other patients. To date they are the only patients with MCAHS3 without any skeletal defects, and also the only patients having neither cardiac nor genitourinary defects (Table 1). Overall, the clinical phenotype of patient 1 and 2 expand the clinical presentation of MCAHS3.

The genotype-phenotype correlation in MCAHS3 needs further exploration. The patients with the homozygous *PIGT* p.Thr183Pro were the most severely affected (Table 1) [11]. We speculate that the aberrant proline perturbed the functionality of the adjacent amino acid, Cys182, which covalently links Cys92 in GPI8, and is important for GPI-TA activity [3]. The remaining identified *PIGT* mutations are compound heterozygous, combining the missense mutation p.Arg488Trp with a null variant (p.Glu84* or p.Val307Argfs*13) [16,17]. These patients exhibited cerebral and cerebellar atrophy and cardiac defects, while other clinical features varied (Table 1). The homozygous *PIGT* p.Gly360Val variant may be more tolerable to the protein function, as glycine and valine are both small and non-polar amino acids, with moderate physiochemical differences. Nevertheless, this variant was shown to cause a pronounced reduction in the level of GPI-anchored proteins on the plasma membrane in peripheral leucocytes and a milder defect in fibroblasts (Figure 2). Similar residual activity of the GPI-TA complex was found in previous patients [11,16,17] (Supplementary Table S2), and total lack of activity is possibly lethal, as homozygous null variants have so far not been reported in *PIGT* or other genes encoding enzymes involved in GPI biosynthesis.

## 5. Conclusions

We report a novel *PIGT* c.1079G>T (p.Gly360Val) variant in two brothers with epileptic encephalopathy, cerebellar degeneration, ophthalmological, and dysmorphic craniofacial features typical for MCAHS3. They also exhibited signs of pyramidal tract dysfunction. However, they did not show skeletal, cardiac, and genitourinary abnormalities. This report expands the phenotypic spectrum of *PIGT* variants, highlights the variable expressivity of the MCAHS3, and suggests that the neurological defects are the core features, while anomalies in other organ systems may not be regarded as pathognomonic of MCAHS3 in the diagnostic process.

## Figures and Tables

**Table 1:** Summary of the main clinical features in the patients with Multiple Congenital Anomalies-Hypotonia Seizures Syndrome 3 (MCAHS3) reported to date. +, clinical feature present; −, clinical feature absent. Abbreviations: CVI, cortical visual impairment; ID, intellectual disability; F, female; GA, gestational age; HC, head circumference; M, male; Mo, moderate; N, normal; NR, not reported; P, patient; Pro, profound; PSF, primitive sylvian fissures; Se, severe.

Reference	Skauli et al.		Lam et al. [16]		Nakashima et al. [17]	Kvarnung et al. [11]			
Patients	1	2	1	2	1	V-1	V-2	V-4	V-5
*PIGT* Variants	c.1079G>T; p.Gly360Val		c.918dupC; p.Val307Argfs*13/c.1342C>T; p.Arg488Trp		c.250G>T; p.Glu84*/c.1342C>T; p.Arg488Trp	c.547A>C; p.Thr183Pro			
Gender	M	M	F	M	F	F	F	F	F
Weeks GA	40	40	<32	<32	40	40	39	37	37
HC > 90th at birth	-	+	−	−	+	+	+	+	+
Progressive neurological features	+	+	+	+	+	+	+	+	+
ID	Se	Mo-Se	Pro	Pro	Pro	Se	Se	Se	Se
Hypotonia	+	+	+	+	+	+	+	+	+
Epileptic seizures	+	+	+	+	+	+	+	+	+
Cerebral and cerebellar atrophy	+	+	+	+	+	PSF	-	+	+
Esotropia	+	+	+	+	+	+	+	+	+
Nystagmus	+	+	+	+	+	+	+	+	+
CVI	+	+	+	+	+	+	+	+	+
Heart defect	−	−	+	+	+	+	-	+	+
Nephrocalcinosis/urolithiasis	−	−	−	−	+	+	+	+	+
Genitourinary abnormalities	-	-	-	-	+	+	+	+	+
Skeletal abnormalities	−	−	+	+	+	+	+	+	+
Pectus excavatum	−	−	+	+	-	+	-	-	+
Scoliosis	−	−	+	+	+	+	+	-	-
Mesomelic shortening upper limbs	−	−	−	−	-	+	+	+	-
Slender long bones	−	−	+	+	-	-	-	-	+
Osteopenia	−	−	+	+	+	+	+	+	-
Bone age	N	N	Advanced	N	NR	Delayed	Delayed	Delayed	Delayed
Low serum alkaline phosphatase	−	−	−	−	+	+	+	+	+
High forehead, frontal bossing, bitemporal narrowing	+	+	+	+	NR	+	+	+	+
Short nose, anteverted nares, depressed nasal bridge	+	+	+	+	+	+	+	+	+
Wide, open mouth	+	+	+	+	+	+	+	+	+
High palate	+	+	+	+	+	NR	NR	NR	+
Inverted nipples	+	+	+	+	+	NR	NR	NR	+

## Supplementary Materials

Supplementary materials can be found at www.mdpi.com/2073-4425/7/12/108/s1, Table S1: List of homozygous recessive and compound heterozygous variants shared by patient 1 and 2 after variant filtering of WES data; Table S2: Summary of glycosylphosphatidylinositol (GPI) linked protein expression in patients with MCAHS3.
