# Supplementary material for: Novel PIGT Variant in Two Brothers: Expansion of the Multiple Congenital Anomalies-Hypotonia Seizures Syndrome 3 Phenotype
Source: Genes (Basel). 2016 Nov 29;7(12):108. doi: 10.3390/genes7120108 (PMC5192484; doi:10.3390/genes7120108)
Supplement: Supplementary file 1 [file genes-07-00108-s001.docx]

Supplementary Materials: Novel *PIGT* Variant in Two Brothers: Expansion of the Multiple Congenital Anomalies-Hypotonia Seizures Syndrome 3 Phenotype

Nadia Skauli, Sean Wallace, Samuel C. C. Chiang, Tuva Barøy, Asbjørn Holmgren,
Asbjørg Stray-Pedersen, Yenan T. Bryceson, Petter Strømme, Eirik Frengen and Doriana Misceo

**Table S1.** List of homozygous recessive and compound heterozygous variants shared by patient 1 and 2 after variant filtering of Whole Exome Sequencing (WES) data.

| **Gene** | **Position (bp)  Hg19** | **Ref** | **Alt** | **Type of Variant** | **Polyphen2 Prediction (Score)** | **SIFT Prediction (Score)** | **Zygosity** | **Depth** |
| --- | --- | --- | --- | --- | --- | --- | --- | --- |
| *OR9A2* | chr7:142723516 | G | GAGA | In frame insertion |  |  | Homozygous | 78 |
| *HEYL* | chr1:40105297 | T | C | Missense | Po.D (0.885) | D (0) | Homozygous | 45 |
| *TMEM125* | chr1:43738427 | C | T | Missense | Po.D (0.615) | D (0) | Homozygous | 127 |
| *GJA9* | chr1:39340858 | CA | C | Frame shift |  |  | Homozygous | 32 |
| *PIGT* | chr20:44050068 | G | T | Missense | Pr.D (1) | D (0) | Homozygous | 106 |
| *MAMLD1* | chrX:149680360 | C | T | Missense | Pr.D (0.98) | D (0.01) | Homozygous | 38 |
| *ZNF208* | chr19:22155321 | C | T | Missense | Pr.D (0.956) | D (0.04) | Homozygous | 117 |
| *OFD1* | chrX:13764978 | T | G | Missense | Pr.D (0.952) | D(0.01) | Homozygous | 12 |
| *CD109* | chr6:74495175 | T | C | Missense | Pr.D (0.973) | D (0) | Homozygous | 33 |
| *COL16A1* | chr1:32163604 | C | T | Missense | Unknown (0) |  | Homozygous | 127 |
| *HIVEP3* | chr1:41976579 | G | A | Missense | Pr.D (0.955) | D (0.01) | Homozygous | 127 |
| *DMD* | chr6:32235149 | G | A | Missense | Po.D (0.488) | D (0.02) | Homozygous | 16 |
| *ZNF492* | chr19:22847163 | G | A | Missense | Po.D (0.436) | D (0.04) | Homozygous | 78 |
| *ZNF492* | chr19:22847487 | G | A | Missense | Po.D (0.794) | D (0.01) | Homozygous | 127 |
| *PCLO* | chr7:82578811 | T | G | Missense | Unknown (0) |  | Heterozygous | 18 |
| *PCLO* | chr7:82764900 | A | C | Missense | Unknown (0) |  | Heterozygous | 40 |
| *NACA* | chr12:57111550 | G | A | Missense | unknown(0) |  | Heterozygous | 19 |
| *NACA* | chr12:57111562 | G | A | Missense | Unknown (0) |  | Heterozygous | 19 |
| *PABPC1* | chr8:101721812 | G | A | Missense | Po.D (0.849) | D (0) | Heterozygous | 228 |
| *PABPC1* | chr8:101721817 | T | C | Missense | Pr.D (0.97) | D (0) | Heterozygous | 225 |
| *PABPC1* | chr8:101721839 | C | A | Missense | Po.D (0.839) | D (0) | Heterozygous | 239 |
| *ATAD3B* | chr1:1412659 | G | T | Missense | Pr.D (1) | D (0) | Heterozygous | 168 |
| *ATAD3B* | chr1:1421954 | G | A | Missense | Pr.D (0.956) | D (0.04) | Heterozygous | 358 |
| *FAM86B2* | chr8:12283473 | C | T | Missense | Unknown (0) |  | Heterozygous | 180 |
| *FAM86B2* | chr8:12286158 | A | T | Missense | Pr.D (0.994) | D (0.01) | Heterozygous | 209 |
| *BPIFA3* | chr20:31812208 | C | A | Nonsense |  |  | Heterozygous | 32 |
| *BPIFA3* | chr20:31813012 | C | A | Nonsense |  |  | Heterozygous | 78 |
| *FAM86B1* | chr8:12041190 | C | T | Missense | Unknown (0) |  | Heterozygous | 169 |
| *FAM86B1* | chr8:12044217 | G | C | Nonsense |  |  | Heterozygous | 179 |

Alt, alternative allele; D, deleterious; Po.D, possibly damaging; Pr.D, probably damaging; bp, base pair; Ref, reference allele.

**Table S2.** Summary of glycosylphosphatidylinositol (GPI) linked protein expression in patients with Multiple Congenital Anomalies-Hypotonia Seizures Syndrome 3 (MCAHS3).

|  |  | | **Granulocytes** | | | | **Monocytes** | | | **B Cells** | | **Fibroblasts** | | |
| --- | --- | --- | --- | --- | --- | --- | --- | --- | --- | --- | --- | --- | --- | --- |
| **Reference** | **Patients** | **Mutation** | **FLAER** | **CD16** | **CD24** | **ALP** | **FLAER** | **CD14** | **CD48** | **FLAER** | **CD24** | **FLAER** | **CD49** | **CD90** |
| Skauli et al. | 1 | c.1079G>T (hmz) | - | - | - | NR | - | - | - | - | - | - | - | - |
|  | 2 | c.1079G>T (hmz) | NR | NR | NR | NR | NR | NR | NR | NR | NR | - | - | - |
| Lam et al. [16] | 1 | c.918dupC (hmz) | - | - | NR | NR | NR | NR | NR | NR | NR |  |  |  |
|  | 2 | c.918dupC (hmz) | - | - | NR | NR | NR | NR | NR | NR | NR |  |  |  |
| Nakashima et al. [17] | 1 | c.250G>T; c.1342C>T | - | - | NS | - | NR | NR | NR | NR | NR |  |  |  |
| Kvarnung et al. [11] | V-1 | c.547A>C (hmz) | - | - | - | NR | NS | NS | NS | NS | - |  |  |  |
|  | V-2 | c.547A>C (hmz) | - | - | - | NR | NS | NS | NS | NS | - |  |  |  |
|  | V-4 | c.547A>C (hmz) | - | - | NR | NR | - | - | NR | NR | NR |  |  |  |

Surface expression of GPI anchored proteins (GPI-Aps) on granulocytes, monocytes, B cells and fibroblasts in patients with MCAHS3 reported to date. Abbreviations: hmz, homozygous; -, decreased compared to healthy control; NS, no significant difference compared to healthy control; NR, not reported.
